# Video frame prediction of microbial growth with a recurrent neural network

**DOI:** 10.3389/fmicb.2022.1034586

**Published:** 2023-01-05

**Authors:** Connor Robertson, Jared L. Wilmoth, Scott Retterer, Miguel Fuentes-Cabrera

**Affiliations:** ^1^Department of Mathematical Sciences, New Jersey Institute of Technology, Newark, NJ, United States; ^2^Department of Environmental Science and Technology, University of Maryland, College Park, MD, United States; ^3^Center for Nanophase Materials Sciences, Oak Ridge National Laboratory, Oak Ridge, TN, United States

**Keywords:** microbial growth, Recurrent Neural Network, deep learning, video frame prediction, machine learning, population growth

## Abstract

The recent explosion of interest and advances in machine learning technologies has opened the door to new analytical capabilities in microbiology. Using experimental data such as images or videos, machine learning, in particular deep learning with neural networks, can be harnessed to provide insights and predictions for microbial populations. This paper presents such an application in which a Recurrent Neural Network (RNN) was used to perform prediction of microbial growth for a population of two *Pseudomonas aeruginosa* mutants. The RNN was trained on videos that were acquired previously using fluorescence microscopy and microfluidics. Of the 20 frames that make up each video, 10 were used as inputs to the network which outputs a prediction for the next 10 frames of the video. The accuracy of the network was evaluated by comparing the predicted frames to the original frames, as well as population curves and the number and size of individual colonies extracted from these frames. Overall, the growth predictions are found to be accurate in metrics such as image comparison, colony size, and total population. Yet, limitations exist due to the scarcity of available and comparable data in the literature, indicating a need for more studies. Both the successes and challenges of our approach are discussed.

## 1. Introduction

Recurrent Neural Networks, RNNs, are a type of Artificial Neural Network that takes a temporal sequence as inputs, learns the spatiotemporal variations, and predicts future data. RNNs were originally created for tasks related to Natural Language Processing, such as text classification and translation, but were subsequently extended to temporal sequences of images (i.e., videos) by incorporating convolutions. These convolutions combine collections of pixels in previous images to predict each individual pixel in the next image of a sequence, allowing the network to capture spatiotemporal dynamics for prediction. RNNs are now used in a variety of applications such as video captioning and video frame prediction (Shi et al., 2015; Su et al., 2020). In biology and microbiology, RNNs have recently been used to study proteins (Alley et al., 2019; Le, 2019; Tng et al., 2022), whole bacteria genomes (Alley et al., 2019), and bacterial growth (Cheroutre-Vialette and Lebert, 2002; Baranwal et al., 2022). For example, in the case of proteins, RNNs can be used on large numbers (millions) of amino acid sequences to learn the statistical representations that effectively approximate the encoded protein features, which does not necessarily require structural or evolutionary data (Alley et al., 2019). On the other hand, in Baranwal et al. (2022) and Cheroutre-Vialette and Lebert (2002) an RNN was used to predict growth curves. In a similar manner, RNNs could be trained on multiple images, rather than multiple amino acid sequences, of bacterial cells as they grow under different conditions. To the best of our knowledge, however, RNNs have not been used to perform video frame prediction of bacterial growth. This is the focus of the work presented here: instead of predicting growth curves, we predict fluorescence microscopy images of growth. It's important to mention that growth curves can be obtained directly from such images by simply counting the number of pixels for each bacteria species.

Over the last few years, new advances have made video frame prediction with RNNs more accurate. A comprehensive review on this matter can be found in Oprea et al. (2020). One such advance has been named predRNN (Wang et al., 2021), which has been successfully used for performing video frame prediction of numbers moving across a screen (the so-called “moving MNIST” dataset ^1^), humans performing different actions (KTH action dataset^2^), traffic flow, and moisture movement in weather systems (Wang et al., 2021). In each of these scenarios, predRNN outperformed previous RNNs. Here we investigate whether predRNN could be used to perform video frame prediction of microbial growth using videos generated by fluorescence microscopy and microfluidics.

**Figure 1:**
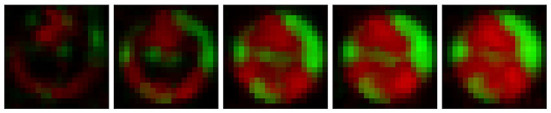
Brightened snapshots at time steps 1, 4, 7, 10, and 13 for one of the videos obtained in Timm et al. (2017) for the 30 μm well. The two mutant strains of *P. aeruginosa*, T6SS-positive and T6SS-negative, appear in green and red, respectively.

Training an RNN to perform video frame prediction requires a large number of videos. For example, predicting the random movement of numbers across a screen required 10,000 videos of 20 frames each (Wang et al., 2021). In microbiology, combining microfluidics with fluorescence microscopy has the potential to produce large amounts of videos. Timm et al. (2017) used microfluidics with fluorescence microscopy to investigate the growth of *Pseudomonas aeruginosa*, an antibiotic resistant bacteria that is responsible for many clinical infections (Pang et al., 2019). Using a photolithographic procedure, this group built a chip consisting of an array of microwells with different diameters. The diameters ranged from 5 to 100 μm, and each well had several replicates to ensure the data collected was statistically significant. The chip was seeded with a mixed microbial population that contained two mutant strains of *P. aeruginosa* at different concentrations. One mutant possessed the Type VI Secretion System, called here T6SS-positive, while the other mutant, T6SS-negative, lacked it, which made it more susceptible to attacks by the T6SS-positive mutant. The T6SS involves a needle-like pilus that some bacteria use to attack others (Sana et al., 2017). To monitor the growth of this mixed population, the T6SS-positive and T6SS-negative mutants were tagged with green and red fluorescent protein, respectively, and their growth monitored with fluorescence microscopy. This microwell fabrication and mixed bacterial population seeding on a chip has opened the door to large scale data-driven analysis of bacterial population interactions (Halsted et al., 2016; Hansen et al., 2016). Five snapshots of a microwell video obtained *via* this procedure are shown in Figure 1.

Here we show that predRNN (Wang et al., 2021) can be used to accurately predict future frames for the microwell videos collected in Timm et al. (2017). We show this by directly comparing predicted and original frames as well as by measuring the population curves and individual colony attributes of the predicted and original frames.

## 2. Methods

Timm et al. (2017) collected videos of microbial growth by generating fluorescence microscopy images of 24 x 24 pixels in RGB (Red, Green, and Blue) format every 30 min for a period of approximately 24 h. In these images, T6SS-positive and T6SS-negative *P*. aeruginosa mutants appeared as green and red, respectively. Of all the well sizes in the micro-fluidic array, we found that the 30 μm well-contained the most useful data. First, this well size allowed for microbial populations to completely fill the well during growth, resulting in a distinct partitioning of the population into colonies of both strains. Second, more videos were collected for this well size than for the other sizes, giving a more complete set for training predRNN. For this reason, we focus the present study on videos collected for the 30 μm wells.

A total of 48 videos of 14 frames were collected for the 30 μm well. In these videos, frames 1–7 showed the most interesting dynamics, which corresponded to the exponential growth of the population from a few initial colonies to a large community that covered the entire well. By contrast, frames 8–14 were practically static, as growth reached a saturation state around frame 8. As a consequence, we discarded frames 8–14 and focused on frames 1 through 7 giving a dataset comprised of 48 videos of seven frames of 24 x 24 pixels in RGB format.

These frames were subjected to a series of four transformations aimed at optimizing the training of the predRNN network. First, we transformed the images from RGB to HSV (Hue, Saturation, Value) format. This transformation aimed to separate the population information contained in each image more clearly than with RGB. Specifically, the presence of either population in a given pixel was recorded by the Value channel (black or not black pixel), the species of the population in the pixel was recorded by the Hue channel (red or green pixel), and the concentration of the species in the pixel was accounted for by Saturation (how bright red or green in the pixel were). Second, each frame was adjusted to match the center of the images with the center of the well. This was done by cropping a square around the brightest pixels of the images averaged over time after triangle thresholding (Zack et al., 1977) which accentuates the brightest pixels in the image. Third, the number of frames per video was increased from 7 to 20 and each frame was expanded from 24 x 24 to 32 x 32 pixels. The number of frames per video was increased using temporal interpolation across frames, and was performed with the scikit-image Python package (van der Walt et al., 2014). The size increase was performed with the bilinear interpolation in the OpenCV library (Bradski, 2000). These three sets of transformations led to a dataset of 48 videos of 20 frames of 32 x 32 pixels recorded in the HSV format. At this point, five of the videos were set aside as a "test" set which would be later used to assess the predictive ability of the neural network on previously unseen data.

**Figure 2:**
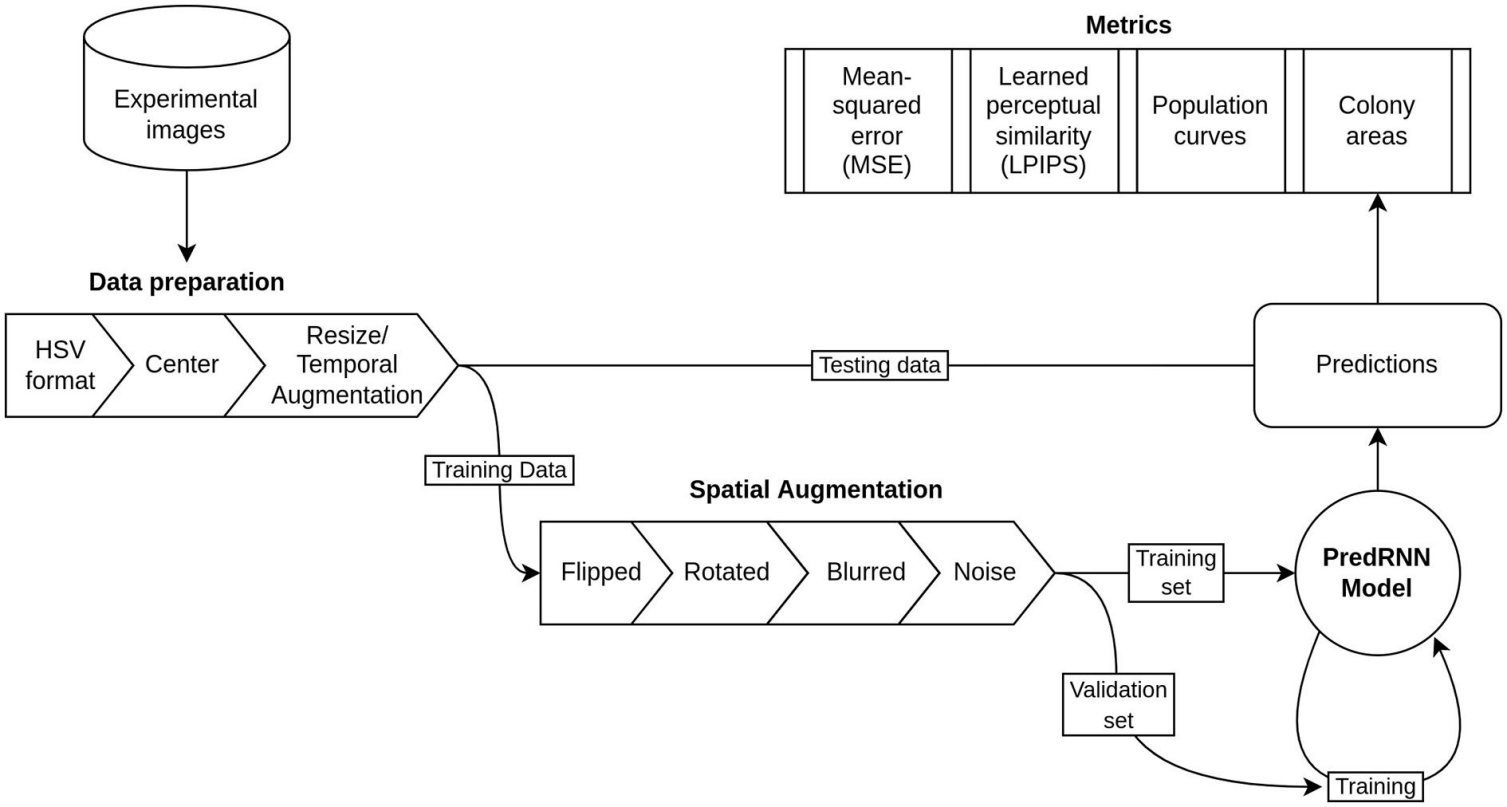
Flow chart of the data and model pipeline including preprocessing steps, dataset separation, and validation metrics.

As the resulting 43 videos still made a relatively small dataset for training predRNN, we performed a fourth set of transformations that consisted of flipping, rotating, blurring, and adding Gaussian noise to all the videos. These transformations were performed with the AtomAI Python package (Ziatdinov et al., 2021) developed for material science applications of deep learning. These final transformations gave a dataset of 392 videos with 20 frames of 32 x 32 pixels in the HSV format. Figure 2 shows schematically the transformations and the workflow for preparing the input dataset.

In order to train the network, the 392 video dataset was randomly split into training and validation sets in a 80/20 proportion which are used in the training process. This is a customary practice in machine learning meant to verify that the model can perform well on new data (i.e., data not used to tune the parameters of the model). Specifically, the training set is used to update the “weights” or parameters of the neural network. The model is then tested on the validation set to get a measure of how well it is performing on new data. The process is then repeated. To assess the final quality of the trained network after training, a test set, which is withheld earlier and never seen by the network during training, is used. This procedure is shown schematically in Figure 2.

During the training procedure, the first 10 frames of a video sequence were used as inputs and the remaining 10 were used for predictions. In what follows, the original 11–20 frames will be referred to as groundtruth (the images we want the network to output), whereas those predicted by the network will be referred to as predicted frames.

There are a variety of network parameters, called hyperparameters, that can be adjusted for improving the training process including size and step of convolutions, parameter normalization, patch sizes of the input images, random sampling of the training data, number of total parameters, learning rate, size of training data batches, and total number of training rounds or “epochs.” Together, these represent over 100 million possible parameter permutations, each of which would require retraining the model (a process that could take hours using a GPU accelerated computer). Due to this prohibitive complexity, we used the standard parameters from the original PredRNN model and varied only the layer size and learning rate. We tested the models performance with each parameter set by iterating across a range of layer sizes and learning weights. To evaluate the success of each set of hyperparameters, we evaluated the mean-squared error (MSE) of the predictions with the groundtruth, which is a pixel-wise comparison of two images and the learned perceptual image patch similarity (LPIPS). The LPIPS metric was recently developed as an image similarity metric to mimic the perception of the human eye (Zhang et al., 2018). It should be noted that the smaller the MSE and LPIPS values are, the more similar the predicted frame is to the corresponding groundtruth. We ultimately determined to use layers of size 32 and a learning rate of 0.0003. A link to *GitHub* repository that contains the trained network, as well as the list of optimal hyperparameter values, is included in the Supporting Information.

## 3. Results and discussion

**Figure 3:**
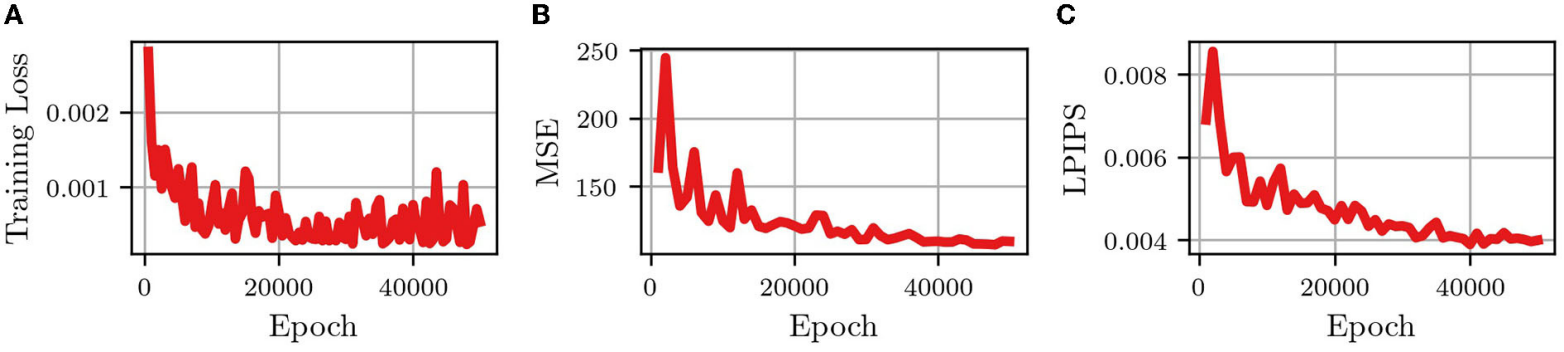
Convergence metrics of predRNN (Wang et al., 2021) during training. **(A)** MSE loss for training images. **(B)** MSE loss for validation images. **(C)** LPIPS loss for validation images.

Figure 3 shows the changes in the training loss (the MSE of “predictions” using the training data) and the MSE and LPIPS of the validation set, with the number of epochs (the number of iterations used to train the network). It is seen that after 50,000 epochs the network had converged to a somewhat steady state in the training loss as well as the MSE and LPIPS validation metrics. This means that when given an input of 10 frames from one of the training videos, the network will accurately output the next 10 frames (good training loss) and when given 10 frames from one of the validation videos the network will output a prediction of 10 frames that are similar pixel-wise (good MSE) and similar by human examination (good LPIPS) to the actual next 10 frames in the video.

**Figure 4:**
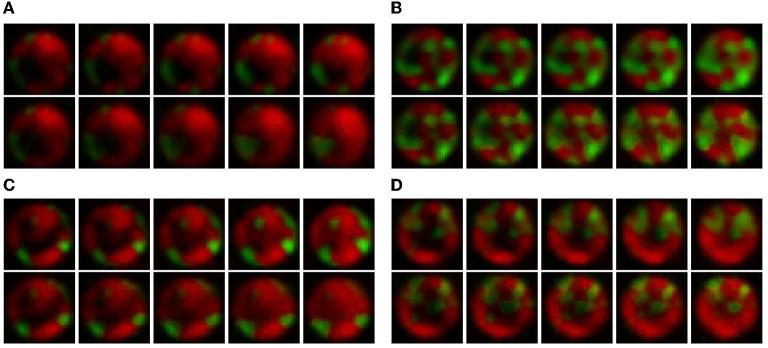
Qualitative comparison between the groundtruth and predicted frames for four wells in the test dataset. In each figure, the upper panel represents the groundtruth frames, and the lower the predicted frames. The images progress from left to right and the wells are numbered according to their position in the test dataset. Data taken from Timm et al. (2017) then expanded in size and interpolated in time as explained in Section 2.

**Figure 5:**
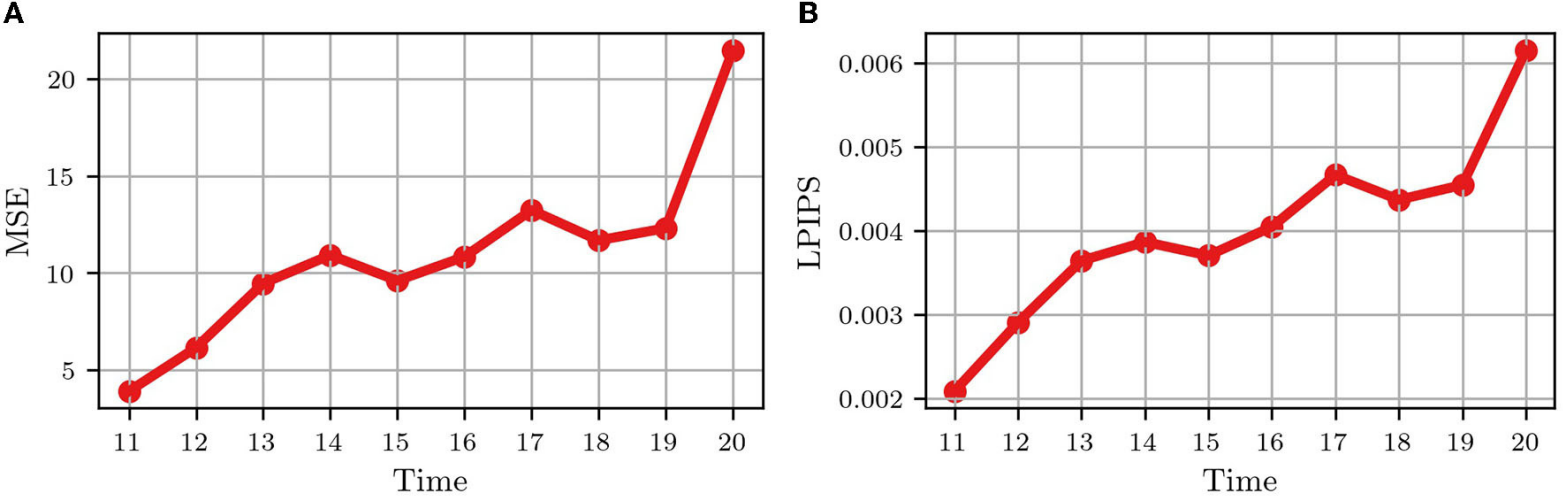
Quantitative comparison between the groundtruth and the predicted frames for the test wells in Figure 1. **(A)** Average mean-squared error (MSE) for all test wells; **(B)** Average learned perceptual image patch similarity (LPIPS) for all test wells. Each dot represents a frame.

Figure 4 shows the video frame predictions for four different wells from the test dataset. It can be observed that the groundtruth frames (upper panel) and the predicted frames (lower panel) are visually very similar. To quantify this similarity, we computed the per-frame and average MSE and LPIPS. Figure 5 shows the per-frame MSE and LPIPS, respectively, and both increase with time. In other words, the predicted and groundtruth frames differ more for predictions performed later in time. This is an expected result because errors accumulate as more frames are predicted. For example, frame 12 is predicted from frame 11, and any error in predicting frame 11 carries on to frame 12. Despite this, the accuracy of the predictions is satisfactory and comparable to the results obtained in Wang et al. (2021) for the moving MNIST database. Further, the average MSE and LPIPS values obtained for the test set are 13.7 and 0.002, which are smaller than the values 48.4 and 0.071 obtained in Wang et al. (2021).

The results above are encouraging as they suggest that video frame prediction of microbial growth can indeed be performed with predRNN. However, aside from directly comparing images, neither MSE nor LPIPS are particularly insightful in the context of microbial experiments. It is instead preferable to compare images with metrics that provide quantitative information on microbial growth and which are commonly used by microbiologists.

**Figure 6:**
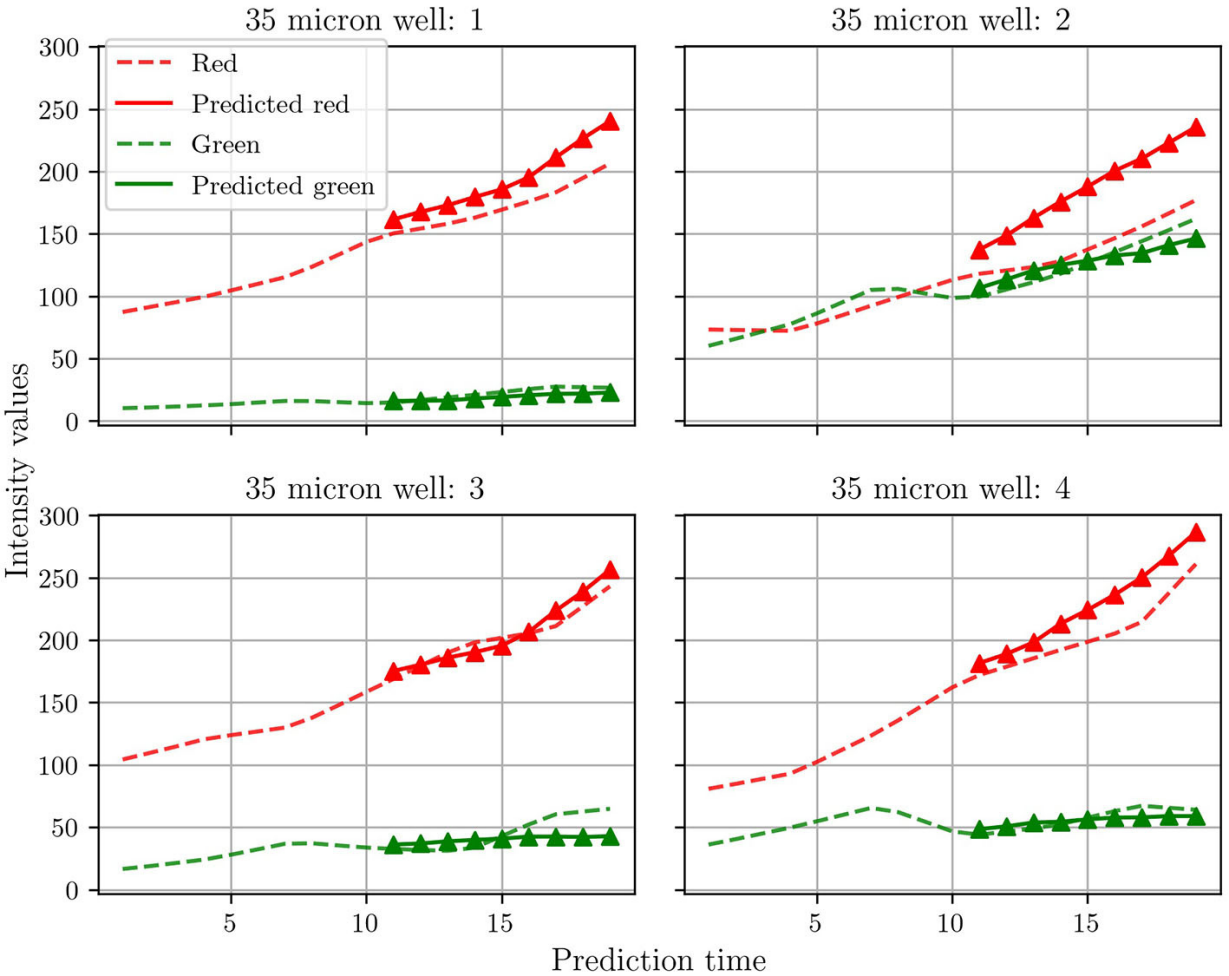
Comparisons between the population curve extracted from the groundtruth frames and the corresponding predicted population curves. See text for an explanation of how the population curves were extracted.

Many previous studies for microbial growth use population growth curves for the respective species as quantitative metrics (Timm et al., 2017). As discussed previously, Timm et al. used green and red fluorescent proteins to tag the T6SS-positive and T6SS-negative mutants of *P. aeruginosa*, respectively. The growth of each mutant, and of the whole community, was then monitored by plotting the intensity of the fluorescent signal vs. time. Here we do not measure the intensity of the fluorescent signal, but instead extract population curves directly from the groundtruth and predicted frames by summing the pixels for green and red. Figure 6 shows the population curves calculated for the four test wells. In general, the agreement between prediction and groundtruth is better for the T6SS-positive (green) than for the T6SS-negative (red) mutant. The reason for this is not entirely clear to us and the scarcity of data has posed a challenge for additional analysis. However, our hypothesis is that as T6SS-positive attacks, the growth of T6SS-negative is more susceptible to fluctuations, which causes predRNN to have more difficulties to learn the spatiotemporal evolution from frame to frame. This can be observed most acutely in the population curves of test well 2 which show an almost equal number of the two strains, causing the most aggressive of fluctuations in population. This particular case is investigated further below. Finally, it is also seen that the T6SS-positive mutant grows less than the T6SS-negative. Understanding the reasons behind this observation is beyond the scope of this work, but some studies have suggested that possessing the T6SS pilus puts a significant energy burden on the T6SS-positive strain, which ultimately limits its growth (Wilmoth et al., 2018).

**Figure 7:**
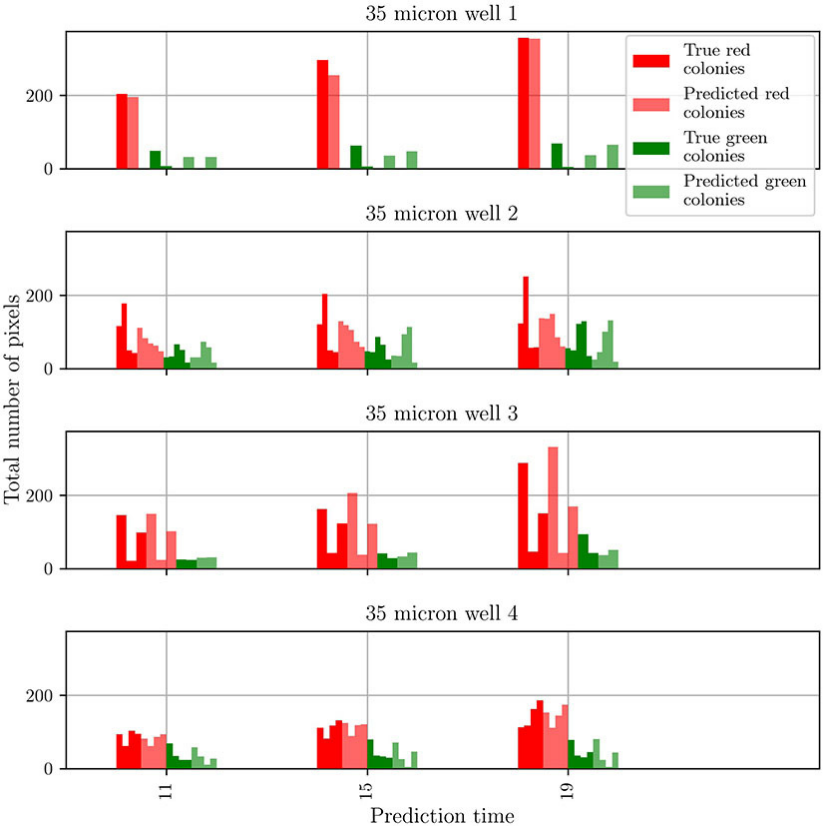
Comparison of the number and size of individual colonies in the groundtruth and predicted frames for wells in the test dataset. The wells are numbered according to their position in the test dataset.

To gain insights into the discrepancy between predictions and observations for test well 2, we extracted two more metrics from the predicted and groundtruth frames. These metrics are the number of colonies for each mutant and the number of pixels comprising each colony. The results obtained for all four test wells are shown in Figure 7. For clarity, the data is shown only for three different frames taken from the whole sequence of groundtruth and corresponding predicted frames. For both mutants, and for wells 1, 3, and 4 the agreement between prediction and groundtruth is in general very good. Minor differences are seen for the T6SS-positive mutant: for well 1, the number of predicted colonies are overestimated, and for well 4, frames 15 and 19 show one colony less for the predicted frame than for the groundtruth one. However, the differences are more significant for test well 2, especially for the T6SS-negative mutant (red). In this case the number of T6SS-negative colonies in the groundtruth and predicted frames, and the corresponding number of pixels in the colonies, are different. On the other hand, for the T6SS-positive mutant, the predictions are very good.

From these results, it is clear that the origin of the differences in the population curves for test well 2 are rooted on differences at the level of individual colonies. To further understand this, we refer to Figure 4: It is seen that, in general, the growth in test wells 1, 3, and 4 is characterized by the partitioning of the population into a large T6SS-negative colony and a few smaller T6SS-positive ones. By contrast, the growth in test well 2 is characterized by the appearance of small colonies for both mutants. It is unknown what might have caused this distinct growth pattern, but visual inspection of the original dataset (48 videos of 14 frames) reveals that this pattern is uncommon. The dataset is thus imbalanced, which leads to a bias in the training of predRNN, hampering its ability to accurately predict the growth for test well 2, specifically for the T6SS-negative mutant. Indeed, for this well the T6SS-positive mutant predictions are accurate because the growth pattern is similar to the patterns observed in other wells. However, for the T6SS-negative mutant, the growth pattern in test well 2 is unusual and the network is not capable of predicting it since it was not trained with many similar cases. Imbalanced datasets are a major yet subtle issue in machine learning, as accurate predictions depend on the specifics of each dataset. In our case, the difficulty is compounded by the scarcity of video data. For example, in this case any effort to balance the dataset by removing videos with uncommon growth patterns would reduce the size of the dataset even further. Finally, our trained model is not expected to accurately predict microbial growth for other well sizes than the 30 μm one, because the training dataset did not include them. To enable that, one would have to retrain the model by including those wells, but unfortunately, as already mentioned, this is not possible due to the scarcity of data.

## 4. Conclusion

We trained an RNN, named predRNN (Wang et al., 2021), to perform video frame prediction of microbial growth for a population containing two mutants of *P. aeruginosa*. For training, we used 48 videos that were previously collected by Timm et al. (2017) with microfluidics and fluorescence microscopy.

To assess the quality of the predictions, we used image-to-image metrics, in particular MSE and LPIPS, as well as microbially significant metrics such as population curves and the characteristics of individual colonies. It was found that predRNN (Wang et al., 2021) can, in general, predict correctly the growth of this population. However, we also noticed that in some cases the predicted growth could be inaccurate. In this particular setting, these inaccuracies were found to be caused by an imbalanced training dataset, which contained more wells with faster T6SS-negative mutant growth than the T6SS-positive mutant. As a consequence, the network tended to favor the growth of the T6SS-negative mutant over the other mutant, which led to large errors in frame prediction for wells where both mutants happened to grow similarly. This emphasizes the need to create large databases of microbial growth, which could be accomplished by combining microfluidics and fluorescence microscopy. A recent study, however, offers a new possibility for increasing the dataset: In Pawlowski et al. (2022) used deep learning style transfer to create a dataset of synthetic images of realistic microbial growth. This technique could be useful for augmenting the dataset for video frame prediction. Nonetheless, despite the challenges associated with the size of the dataset, our results provide promising steps toward the possibility of performing autonomous experiments in microbiology, where time spent in image acquisition could be saved by predicting microbial growth with RNNs.

The successful application of this recurrent model architecture for the included fluorescence images also suggests future applications in predictions of cell morphology such as shape and size (Li et al., 2021; Way et al., 2021), in different spatiotemporal resolutions, or in predicting compositions of more heterogeneous mixtures of diverse bacterial strains (e.g., each strain could be labeled with a unique fluorescent protein; Larsen et al., 2015). Although these applications lie within the scope of the predRNN model architecture, successful predictions would require training using the a large dataset of high resolution images with consistent time and space scales (see Supplementary material for more details on this limitation). For example, to predict cell morphology changes, we could follow the same procedure as documented in this manuscript with high resolution images of cell populations rather than fluorescence microscopy images of populations. The metrics of success would also need to be adjusted to measure characteristics of cell shape or size rather than population or colony level measurements. Each of these applications promise a new range of machine learning based predictive analytics beyond the scope of current quantitative image analysis methods.

## Licences and permissions

This article has been authored by UT-Battelle, LLC under Contract No. DE-AC05-00OR22725 with the U.S. Department of Energy. The United States Government retains and the publisher, by accepting the article for publication, acknowledges that the United States Government retains a non-exclusive, paid-up, irrevocable, world-wide license to publish or reproduce the published form of this article, or allow others to do so, for United States Government purposes. The Department of Energy will provide public access to these results of federally sponsored research in accordance with the DOE Public Access Plan (http://energy.gov/downloads/doe-public-access-plan).

## Data availability statement

The original contributions presented in the study are included in the article/Supplementary material, further inquiries can be directed to the corresponding author.

## Author contributions

MF-C designed the research. CR trained the recurrent neural network and analyzed the results. JW and SR provided and explained the video data. All authors contributed to writing and editing the manuscript. All authors contributed to the article and approved the submitted version.

## Footnotes

^1^MNIST is the Modified National Institute of Standards and Technology database of handwritten digits. It is commonly used to test neural network classification performance.

^2^KTH action is a dataset made up of videos of people moving created by KTH Royal Institute of Technology in Sweden. It is commonly used for testing neural networks in video prediction.
