# Supplementary material for: Video frame prediction of microbial growth with a recurrent neural network
Source: Front Microbiol. 2023 Jan 5;13:1034586. doi: 10.3389/fmicb.2022.1034586 (PMC9850103; doi:10.3389/fmicb.2022.1034586)
Supplement: Supplementary file 1 [file Data_Sheet_1.pdf]

frontiers

Supplementary Information

Correspondence*:
Miguel Fuentes-Cabrera
fuentescabma@ornl.gov

1 SOFTWARE & DATA

The videos, scripts to trained predRNN(Wang et al., 2021), and the trained model can be found in this *GitHub* repository: `https://github.com/miguel-fc/RNNmicrobial`.

2 GENERALIZATION TO NEW DATA

PredRNN represents a spatiotemporal neural network whose predictions rely on consitent time and space scales in the training data. This makes the network's performance on data from wells of different sizes uncertain.

To demonstrate the challenge of generalization to other well sizes (such as might exist in other experiments), we tested the network's performance in predicting the growth of the same mutant *P. aeruginosa* strains in 35 micron wells from the same experimental work of Timm et al. Timm et al. (2017) (as opposed to the 30 micron wells used in the body of this work). A comparison of predictions with the experimental results for several wells (seen in Fig. 8) demonstrates that the although the predictions are fairly reasonable, the network generally predicts more growth than was observed in the experiment. This can be attributed to the increased spatial scale of the 35 micron wells in that each pixel now represents more area than was represented in the training images. i.e. if the model was trained to see an average growth rate of 2 pixels/minute from 30 micron well data, applying this same rate to 35 micron well data will overpredict the rate of the growth of the populations.

The visually observed growth from Fig. 8 can be further validated by observation of the global population curves (Fig. 9) and colony size chart (Fig. 10) for predictions using this new well size. Both metrics demonstrate predictions with a general tendency toward larger growth than observed.

Improving the model's ability to generalize to new scales in time and space will require using a training set of mixed scales (although this will likely decrease the accuracy for any one set of scales) or the use of transformers as preprocessors which can reduce any inputted data to consistent time and space scales. The same two methods could also be applied to allow for generalization to other strains or label colors.

REFERENCES

Timm, A. C., Halsted, M. C., Wilmoth, J. L., and Retterer, S. T. (2017). Assembly and Tracking of Microbial Community Development within a Microwell Array Platform. *JOVE-JOURNAL OF VISUALIZED EXPERIMENTS* doi:10.3791/55701

Wang, Y., Wu, H., Zhang, J., Gao, Z., Wang, J., Yu, P. S., et al. (2021). Predrnn: A recurrent neural network for spatiotemporal predictive learning. *CoRR* abs/2103.09504

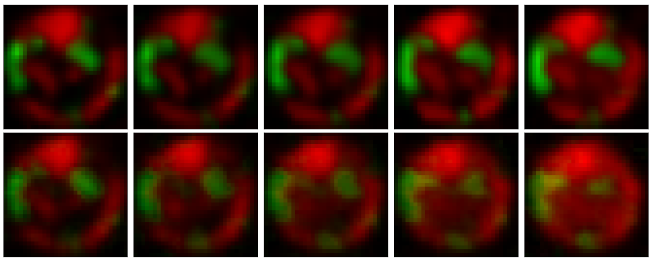

(a) 35 micron well 1

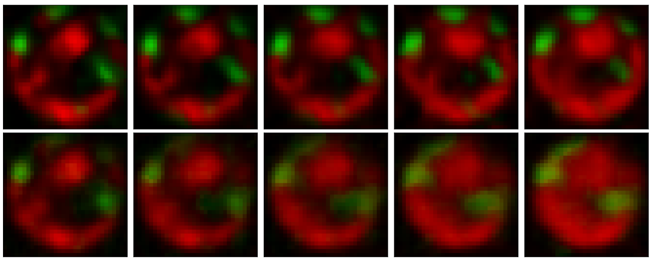

(b) 35 micron well 2

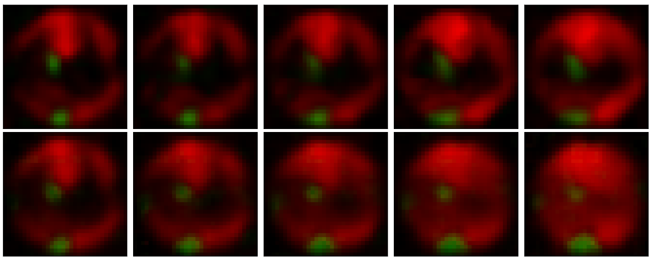

(c) 35 micron well 3

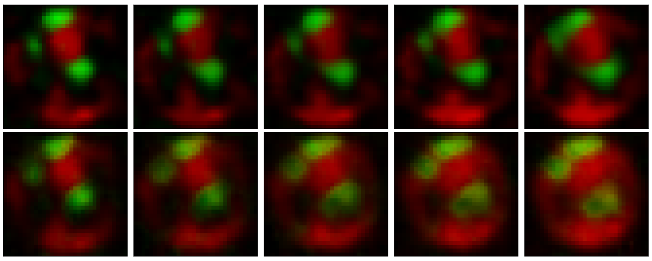

(d) 35 micron well 4

Figure 8. Qualitative comparison between the groundtruth and predicted frames for 35 micron wells. In each figure, the upper panel represents the groundtruth frames, and the lower the predicted frames. The images progress from left to right and the wells are numbered according to their position in the 35 micron test dataset. Data taken from (Timm et al., 2017) then expanded in size and interpolated in time in the same form as the 30 micron wells.

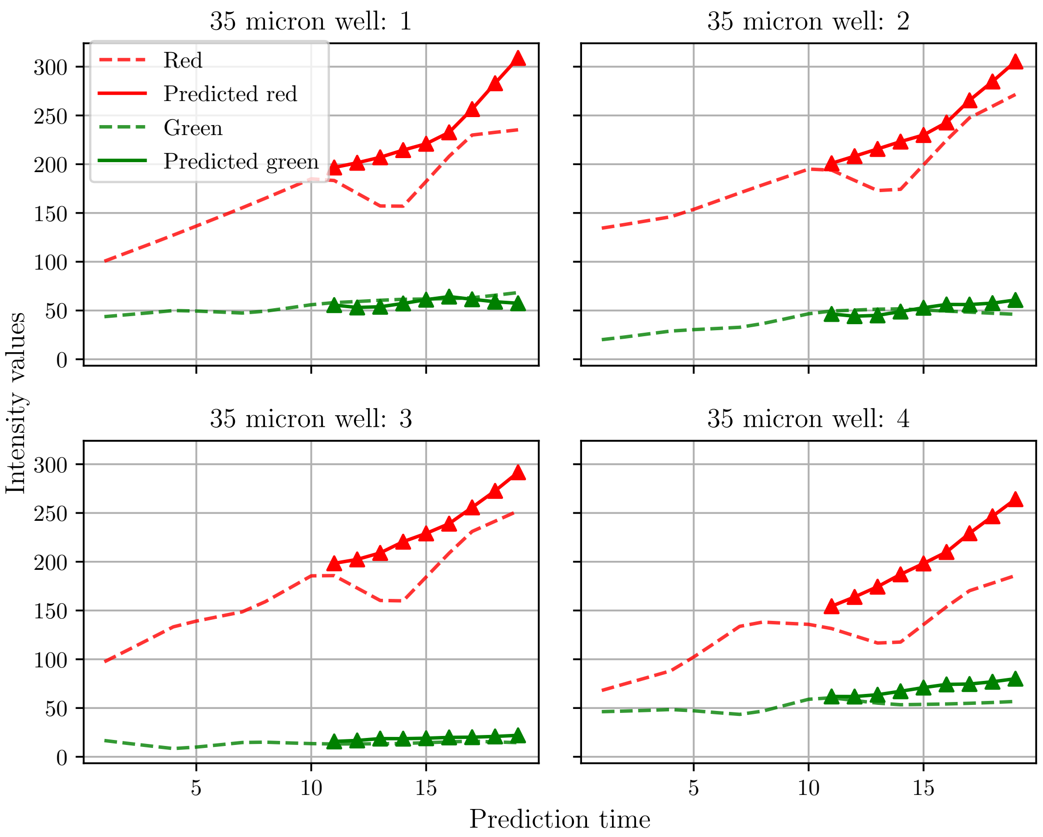

Figure 9. Comparison of the predicted and groundtruth population curves for 35 micron wells.

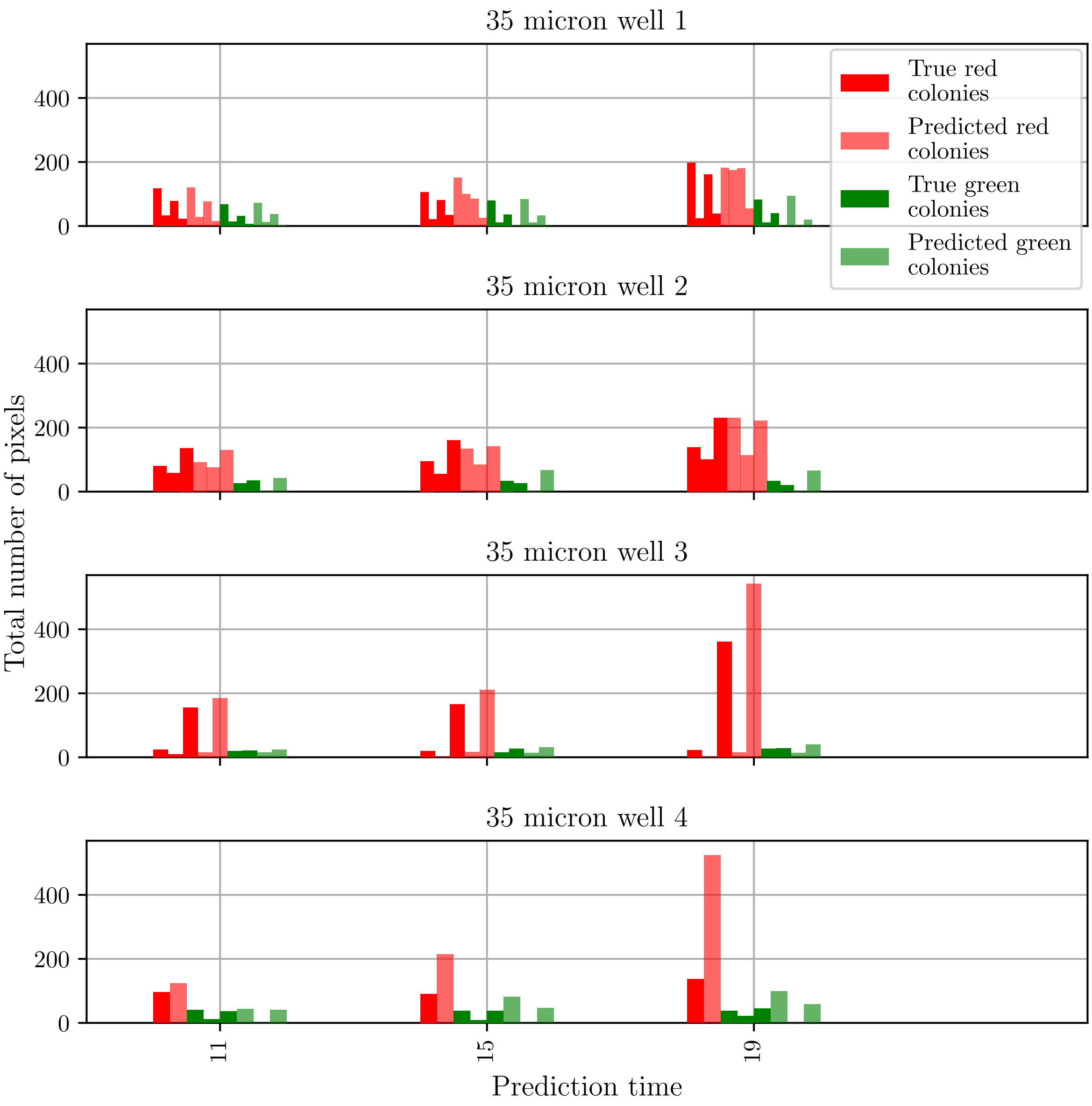

Figure 10. Comparison of the number and size of individual colonies in the groundtruth and predicted frames for 35 micron wells.
